# The Mediterranean Plastic Soup: synthetic polymers in Mediterranean surface waters

**DOI:** 10.1038/srep37551

**Published:** 2016-11-23

**Authors:** Giuseppe Suaria, Carlo G. Avio, Annabella Mineo, Gwendolyn L. Lattin, Marcello G. Magaldi, Genuario Belmonte, Charles J. Moore, Francesco Regoli, Stefano Aliani

**Affiliations:** 1 CNR-ISMAR, Pozzuolo di Lerici - La Spezia, Italy; 2 Instituto Español de Oceanografía, Centro Oceanográfico de Baleares, Palma de Mallorca, Spain; 3 Dipartimento di Scienze della Vita e dell’Ambiente (DiSVA), Universita’ Politecnica delle Marche, Ancona, Italy; 4 Algalita Marine Research and Education, Long Beach, California, USA; 5 Department of Earth and Planetary Sciences, Johns Hopkins University, Baltimore, MD, USA; 6 Universita’ del Salento, DiSTeBA, Lecce, Italy; 7 CoNISMa, Consorzio Interuniversitario per le Scienze del Mare, Roma, Italy

## Abstract

The Mediterranean Sea has been recently proposed as one of the most impacted regions of the world with regards to microplastics, however the polymeric composition of these floating particles is still largely unknown. Here we present the results of a large-scale survey of neustonic micro- and meso-plastics floating in Mediterranean waters, providing the first extensive characterization of their chemical identity as well as detailed information on their abundance and geographical distribution. All particles >700 μm collected in our samples were identified through FT-IR analysis (n = 4050 particles), shedding for the first time light on the polymeric diversity of this emerging pollutant. Sixteen different classes of synthetic materials were identified. Low-density polymers such as polyethylene and polypropylene were the most abundant compounds, followed by polyamides, plastic-based paints, polyvinyl chloride, polystyrene and polyvinyl alcohol. Less frequent polymers included polyethylene terephthalate, polyisoprene, poly(vinyl stearate), ethylene-vinyl acetate, polyepoxide, paraffin wax and polycaprolactone, a biodegradable polyester reported for the first time floating in off-shore waters. Geographical differences in sample composition were also observed, demonstrating sub-basin scale heterogeneity in plastics distribution and likely reflecting a complex interplay between pollution sources, sinks and residence times of different polymers at sea.

Global production of plastic materials increased twenty-fold in the last fifty years, exceeding 300 million tonnes in 2015^1^. Demand is growing exponentially and production is expected to quadruple by 2050, taking up 20% of total oil consumption and 15% of the global carbon budget^2^. Single-use packaging applications represent the largest share of the European plastic market, accounting for 40% of the total production^1^ and for more than 10% of the municipal solid waste^3^. As a result, 275 million tonnes of plastic litter were generated in 2010 by the world’s coastal countries, of which 4.8 to 12.7 million tonnes were estimated to have ended up in the oceans, a scenario projected to increase by an order of magnitude within 2025^3^.

Since the first reports on the presence of plastic in the oceans, worldwide attention has been focussed to this problem and the number of scientific publications has seen an exponential growth^4^. Vast accumulation areas have been discovered in all main oceanic gyres^5^,^6^,^7^,^8^, and every part of the oceans examined so far, revealed the presence of marine litter, including polar seas^9^ and deep-sea sediments^10^. Plastic is now so abundant that it has been proposed as a new stratigraphic indicator of Anthropocene^11^.

Over 92% of all plastic items found at sea are generally smaller than 5 mm^12^. These tiny particles – hereinafter referred to as microplastics – may either result from the breakdown of larger objects, or they can directly enter the marine environment as granules, pellets and fibers. Microplastics can act as dispersal vectors of chemical additives, organic and metal pollutants accumulated from surrounding waters^13^ and provide habitats for a wide range of rafting organisms and microbial communities^14^. Being in the same size class of many planktonic organisms, ingestion of microplastics is widely reported and the first evidences of trophic transfer are emerging^15^,^16^. As a result, indications of negative effects at both, individual and sub-organismal levels are starting to appear, to the extent that the classification of plastic waste as hazardous was recently suggested^17^.

Global models consistently predict some of the highest concentrations of floating plastics in the world to occur in the Mediterranean Sea^12^,^18^, to the extent that together with the main five oceanic gyres, it has been proposed as the sixth great accumulation zone for marine litter^19^. Mainly owing to limited outflow of surface waters, a densely populated coastline and intensive fishing, shipping, touristic and industrial activities, substantial amounts of marine litter are accumulating in the Mediterranean basin, which according to the most recent simulations is retaining between 21% and 54% of all plastic particles (i.e. between 3.2 and 28.2 × 10^12^ particles) and between 5% and 10% of the global plastic mass (i.e. between 4.8 and 30.3 thousand tonnes)^20^.

Mediterranean biodiversity is certainly not immune from interaction with marine litter^21^. Artificial polymers have been found in the stomach contents of Mediterranean pelagic predators^22^, deep-sea fishes^23^ and commercial species^24^. Large amounts of plastic debris have been reported from the Mediterranean seafloor^25^ and floating on its surface^26^, as well as on beaches^27^,^28^ and coastal environments^29^,^30^. Studies on the abundance of floating micro-sized particles however, mostly focused on the NW part of the basin (Table 1) and the polymeric identity of such floating particles is still largely unknown.

The European Marine Strategy Framework Directive (2008/56/EC) highlighted concerns for the environmental implications of marine litter and underlined the urgent need for member countries to “Determine trends in the amount, distribution and composition of micro-particles (mainly microplastics) in European waters and to establish baseline quantities, properties and potential impacts”^31^. More recently, the importance of this issue has also been acknowledged by the Contracting Parties to the Barcelona Convention and by the G7 world leaders, who committed to a global action plan to combat marine litter^32^. Most of the surveys conducted so far however, mainly relied on the visual identification of particles, or characterized only a restricted subset of samples. Thus, detailed information on the actual polymeric diversity of these emerging pollutant is lacking.

Within this context, we present the results of a large-scale survey of micro (<5 mm) and meso-plastics (5–20 mm) occurrence in central-western Mediterranean waters, providing the largest polymeric characterization of floating microplastics ever performed (n = 4,050 particles). In agreement with numerical predictions, we confirm the Mediterranean Sea as severely contaminated by plastic pollution and describe for the first time the complex mixture of synthetic polymers floating on its surface. We tested the hypothesis that plastic distribution and composition are not homogeneous and that geographical differences exist between Mediterranean sub-basins. Such information is urgently required to identify sources and sinks of these emerging pollutants and to better understand fate and impacts of different polymers in the marine environment, so that knowledge-based reduction and prevention measures can be effectively implemented.

## Results

A total of 74 neuston samples were collected during the survey (Fig. S1). Plastic-like particles were found in all samples with a mean total abundance of 1.25 ± 1.62 particles/m^2^ (see Table S1 for other units of measurement). Most of these particles (93.2%) were visually classified as irregularly shaped fragments, while pellets, films and foams constituted only a small fraction of the total (2.2%, 1.6% and 3.1% respectively). Due to the high risk of external contamination, all fibers and filaments were removed from our dataset and not considered in density calculations.

**Figure 1:**
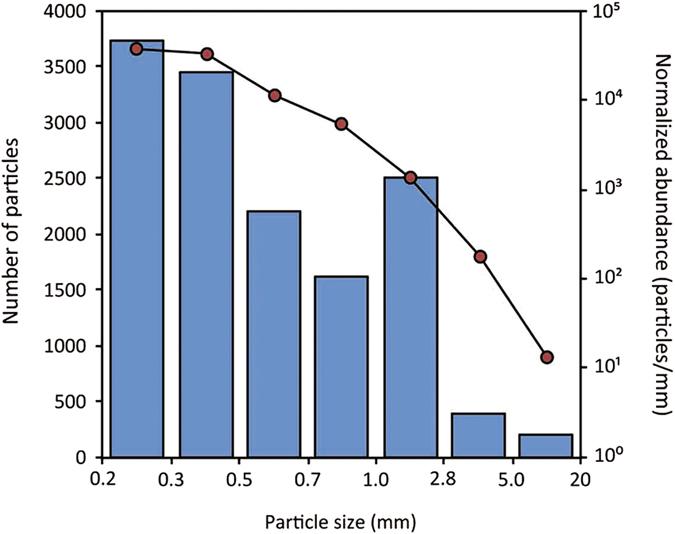
Size distribution of all particles collected during the survey. Normalized abundance values (red dots) were obtained by dividing the total number of particles counted in each size class (blue bars) by the width of the respective size bin expressed in millimeters^8^ (n = 14,106). Please note that fibers and filaments were not included and that only particles >700 μm were characterized through FT-IR spectroscopy. Secondary vertical axis is in logarithmic scale.

The overall size-class distribution revealed a marked prevalence of smaller particles (Fig. 1). 26% of all counted particles were smaller than 300 μm and 51% were smaller than 500 μm (n_*tot*_ = 14,106 particles). Only 197 items (1.4% of the total) were larger than 5 mm. The mean abundance of these meso-particles was very low (0.016 ± 0.028 particles/m^2^) but strongly correlated to the abundance of micro-particles (*r*_*s*_ = 0.8, *p* = 3.6118E-18).

**Figure 2:**
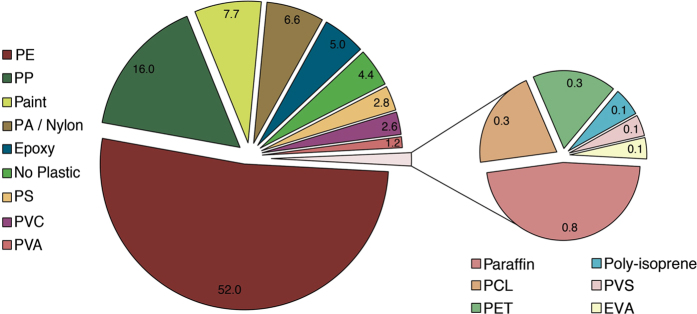
Polymeric composition of all particles >700 μm characterized through ATR FT-IR analysis (n = 4,050 particles). Values are expressed in percentages. Identification of polymers was performed by comparison with a library of standard spectra and only polymers matching reference spectra for more than 60% were accepted.

The polymeric identity of all particles >700 μm was verified through ATR FTIR (n = 4,050 particles). This subset was considered highly representative since it comprised 96.2% of the total weight of collected material. 16 different polymer typologies were identified (Fig. 2 and Fig. S3). Polyethylene (HD-PE and LD-PE) was the predominant form with an overall frequency of 52%, followed by polypropylene (PP) (16%) and synthetic paints (7.7%). Polyamides (PA) accounted for 4.7% of all characterized particles (excluding nylon which accounted alone for 1.9%), whereas polyvinyl chloride (PVC), polystyrene (PS) and polyvinyl alcohol (PVA) represented 2.6%, 2.8% and 1.2% respectively. Other less frequent polymers (<1%) included: poly(ethylene terephthalate) (PET), polyisoprene (synthetic rubber), poly(vinyl stearate) (PVS), ethylene-vinyl acetate (EVA) and cellulose acetate. Ten fragments of polycaprolactone, a biodegradable polymer, were found in seven different samples throughout the study area, while 201 fragments of epoxy resin (polyepoxide) were collected at a single location in the Balearic Sea. Similarly, several residues of paraffin wax were found exclusively in an off-shore sample in the Adriatic Sea. The molecular characterization also revealed a relatively low misidentification rate during visual sorting. Only 4.4% of all analyzed particles did not consist of plastic but were rather made of cotton, chitin, cellulose and other non-synthetic materials.

Because we could not be sure about the synthetic nature of particles <700 μm (see methods and section below), abundances were computed for two additional sub-categories, i.e. particles bigger and smaller than 700 μm, resulting in two mean concentrations of 0.40 ± 0.74 and 0.85 ± 0.95 particles/m^2^ respectively (Fig. S2 and Table S1). Yet, the number of particles counted in each of these two categories, exhibited a strong positive correlation (r_*s*_ = 0.8233, *p* = 2.2163E-19), suggesting a link between size classes and slightly enhancing the level of confidence in our detection rates.

**Figure 3:**
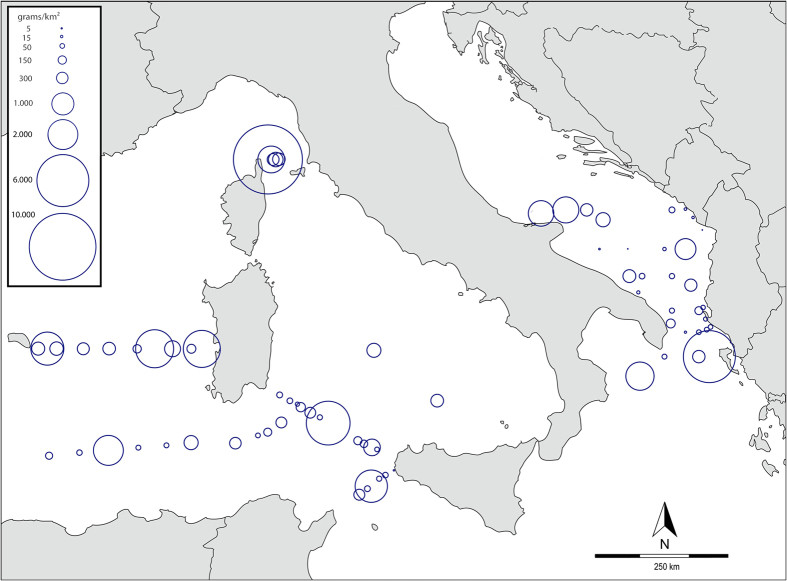
Map of the central-western Mediterranean Sea showing the location of all sampling stations and the distribution of un-corrected plastic densities expressed as grams of plastic per km^2^. Size of the circles is proportional to measured concentration values on a logarithmic scale. Particles <700 μm and synthetic fibers were not included in density calculations. Data were plotted using GPS Visualizer (http://www.gpsvisualizer.com/) and post-edited in Adobe Illustrator CS5. Background map freely retrieved from DEMIS OpenGIS Web Map Server under open copyright licence (http://www.demis.nl/home/pages/wms/docs/OpenGISWMS.htm).

In terms of weight, the amount of particles >700 μm, whose synthetic nature was verified through FT-IR spectroscopy, showed a very high spatial heterogeneity spanning two or three orders of magnitude across the study area (Fig. 3). The maximum value (10.43 kg/km^2^) was observed in the Corsica Channel, between Cap Corse and Capraia island, while the two lowest concentrations were found in the southern Adriatic Sea. Overall, plastic was significantly (*p* = 0.002) less abundant in the Adriatic Sea (467.79 ± 1133.88 g/km^2^; *n* = 30) than in western Mediterranean samples (811.08 ± 1769.75 g/km^2^; *n* = 44). A mean total density of 671.91 ± 1544.16 g/km^2^ was found throughout the study area. Yet, two samples accounted together for one third of the collected plastic mass (34%). When removing these two samples from the dataset, the mean total concentration dropped to 463.49 ± 823.52 g/km^2^. A first-order approximation of the total mass of plastic floating in Mediterranean waters was obtained by computing 95% BCa bootstrapped confidence intervals of our mean density value averaged over the surface of the entire basin (2.5 × 10^6^ km^2^). This resulted in a total estimated load ranging from 873.55 to 2576.03 tonnes of plastic.

**Figure 4:**
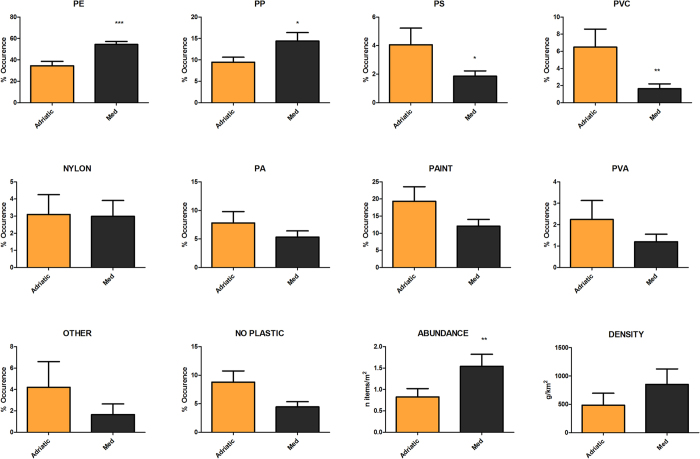
Differences between Adriatic (orange) and western Mediterranean samples (black) in the relative frequencies of the most common types of polymers identified through FT-IR analysis (n = 4,050 particles). Differences in the mean total abundance (items/m^2^) and density (g/km^2^) of particles collected are also shown in the last two panels.

**Figure 5:**
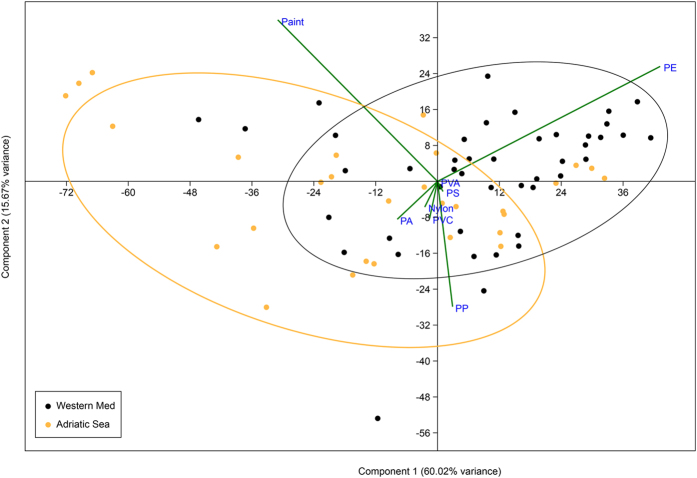
PCA ordination based on the occurrence of the seven most common polymer typologies. All 74 samples are plotted in relation to the first two principal components (PC1 and PC2). Orange dots represent Adriatic samples (n = 30) while western Mediterranean samples are in black (n = 44). Distance biplot of the eigenvectors (not in scale with data points) is shown to help with the interpretation of the loadings results.

Geographical differences between sub-basins were found also in the relative occurrence of different polymers (Fig. 4). The composition of western Mediterranean samples was dominated by low-density polymers such as polyethylene and polypropylene. Adriatic samples instead were more heterogeneous and rather characterized by a higher presence of paint chips, PS, PVC, PVA and PAs. PCA ordination of samples produced a two dimensional pattern, with the first two components explaining 75.7% of the total variance (Fig. 5). Despite some overlapping, most of the separation between sub-basins occurred along PC1 axis, mainly referring to PE (0.80) and paint (−0.57). On the other side, PP (−0.52) determined most of the separation along PC2.

**Figure 6:**
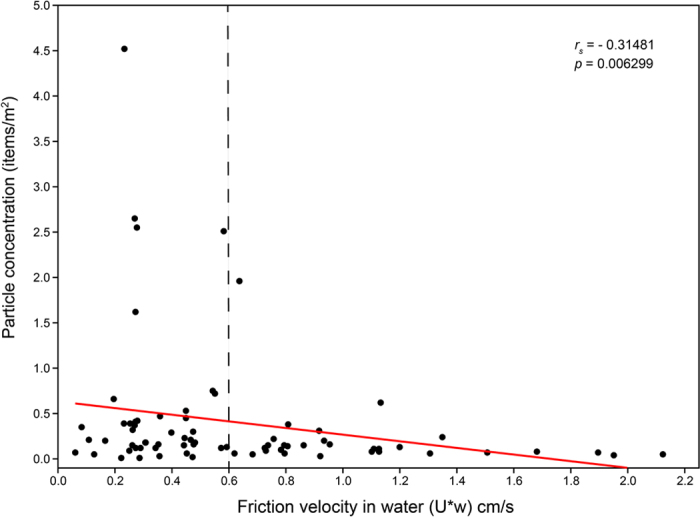
Effect of wind-forcing on the concentration of particles >700 **µm**. Wind-induced friction velocities in water (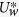) were computed from wind stress (*τ*) values extracted from ERA-Interim model using density of seawater *ρ*_*w*_ = 1.026 g/cm^3^. Spearman’s correlation coefficient (*r*_*s*_) and *p* value are shown in the top-right corner (n = 74). The dashed line corresponds to 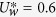 cm/s, above which wind correction was applied^56^.

When testing for the effect of environmental variables on the abundance of particles >700 μm, no significant correlation was found with surface temperature or salinity (*p* > 0.05). Conversely, wind stress and particle concentration were negatively correlated (*r*_*s*_ = −0.31481, *p* = 0.0062991), with high densities being found only at relatively low wind speeds (Fig. 6). When correcting the abundance of particles >700 μm for the effect of wind-induced mixing, a mean correction coefficient of 2.06 was obtained (max 8.97), resulting in an increased average concentration of 0.61 ± 0.94 particles/m^2^ throughout the study area.

## Discussion

The proportion of different polymers found in our study roughly corresponds to the global production stocks of plastic materials, with polyolefins (PE and PP) accounting for 62% of the global plastic demand^33^ and for 68% of our sampled particles. Being widely used in the disposable packaging industry and having lower densities than seawater, it is not surprising that these polymers consistently account for the majority of the plastic particles floating in surface waters worldwide^34^,^35^,^36^. Also being less susceptible to sinking, the contribution of these low-density polymers has been shown to increase with distance from land^37^, hence potentially representing a proxy of the sample’s distance from pollution sources. From this perspective, the higher heterogeneity in the polymeric composition of Adriatic samples, together with an higher occurrence of high-density polymers, would indicate shorter residence times of particles at sea and a closer proximity to pollution sources, likely reflecting the distinctive hydrological features of the Adriatic basin.

The presence of paint and paraffin wax for instance, seems to suggest an high influence of ship-based pollution in the Adriatic Sea. Paint chips are typically generated during the ongoing repair, maintenance and cleaning of vessel decks and hulls^38^ and large quantities of synthetic paints have been recently related to intense marine traffic and shipping activities in Korean waters^39^. Paraffin wax on the other hand, is used for insulation and impregnation, as corrosion protective, additive to rubber products and in cosmetics and candle production. Large quantities are transported by cargo ships and tank-washing residues may be legally discharged at sea beyond the 12 nautical mile zone, including MARPOL designed “special areas” such as the Mediterranean Sea^40^. Paraffin clumps have been previously reported along the coasts of the North and Baltic Sea and in the stomach contents of northern fulmars^41^,^42^, nevertheless its presence in Mediterranean waters had never been explicitly recorded before.

Polycaprolactone (PCL), is a petroleum-based polyester commonly used in 3D printing, hobbyists and biomedical applications. It is considered biodegradable in terrestrial environment, with a degradation time of 6–12 days in laboratory conditions^43^, while some some signs of degradation appeared after 12 months in the marine environment^44^. PCL fragments were found in 9.5% of our net tows throughout our survey area and its presence in Mediterranean off-shore waters provides further evidence that some “biodegradable plastics” do not readily degrade in natural conditions, thus not representing an *a priori* solution for reducing marine litter^33^,^45^.

The range of synthetic materials found in our samples included several polymers – such as PVC, PVA, Paints and PET – whose densities are higher than many solutions commonly used for the extraction of microplastics from the organic matrix^34^,^46^. Although the mechanism through which high-density polymers can persist on the sea surface have yet to be clarified^37^,^47^,^48^, we provide empirical confirmation that, unless heavier solutions are used, such as zinc chloride or sodium iodide, the use of density gradient separation methods can result in a strong underestimation of many polymers commonly encountered in marine surface waters.

Despite general consensus about the loss of particles smaller than 1–2 mm from the sea surface^8^,^19^,^49^,^50^,^51^, we have found no clear indication about this process (Fig. 1), similarly to what was recently reported in the Atlantic Ocean^37^. Certainly without chemical identification of particles smaller than 700 μm we could not be sure about the synthetic nature of our smallest fractions, especially because a growing body of evidences is showing that the error rate during visual sorting noticeably increases with decreasing particle size^34^,^46^,^52^. However the strong correlation found between the abundance of characterized vs non-characterized particles together with a relatively low misidentification rate of 4.4% for characterized particles, seem to suggest good detection rates also for smaller particles, thus slightly enhancing the reliability of our density estimates.

Even considering only particles >700 μm, our measured concentration values are substantially higher than most studies previously performed in the Mediterranean Sea (Table 1). Nevertheless, because the larger size fractions always account for most of the weight, a closer agreement with previous studies is obtained when comparing plastic densities expressed in terms of mass concentrations, rather than particle counts. After removing two outliers from our dataset for instance, our mean density drops to 463.5 g/km^2^, which is incredibly similar to the values reported from the inner accumulation zones of all main oceanic gyres^8^ and to the values of 423 g/km^2^ and 579.3 g/km^2^ obtained in two previous large-scale surveys of the Mediterranean Sea^19^,^53^.

Among the various factors that can explain differences between studies, the way of computing tow distances is likely playing a major role. As a matter of fact, preliminary observations from a recent cruise showed that tow distances calculated through GPS positions are on average twice longer than those calculated using the flowmeter, with this critically resulting in halved plastic abundances (unpublished data). Since the flowmeter’s rotor revolutions measure the effective volume of water passing through the net, taking into account also any eventual clogging, flow-back, transversal currents and vertical movements, GPS may be a less reliable way of calculating the actual sampled area.

Although relatively high plastic concentrations were observed in the Balearic Sea, which was already indicated as a potential short-term retention area by numerical models^54^, no clear accumulation pattern and a high small-scale variability in plastic abundance and composition emerged from our survey, similarly to what was previously reported in the same area for floating macro-debris^26^. In this respect, the formation of permanent accumulation zones in the Mediterranean Sea is realistically hampered by the highly dynamic character of the surface circulation^19^,^54^, of which the observed heterogeneity in plastic distribution is very likely a reflection.

Our reported wind-mixing correction factor (mean 2.06; max 8.97) is very similar to those previously found in the Mediterranean Sea (mean 1.55; max 11.00)^55^, North Atlantic (mean 2.50; max 27.00)^56^ and Australian waters (mean 2.80; max 10.00)^35^. Wind-correction however, should be treated with great care, especially because of the considerable number of approximations and assumptions inherently related to the density of seawater, the buoyant rise speeds of the different polymers, sizes and shapes and to the reliability of modeled wind and wave fields. For these reasons, we preferred to present un-corrected density values in all tables and figures throughout the text.

Lastly, although disparity between numerical models and field observations has been often highlighted, when considering all our particles putatively identified as plastics, our mean total estimate for the whole Mediterranean (3.1 × 10^12^ particles) is in striking agreement with one of three very recent interpolations of ocean circulation models with measured plastic concentrations (3.2 × 10^12^ particles), but substantially lower than the two other predictions^20^. Thus, the goal of future research should be to better understand fate, sinks and fragmentation patterns of different polymers at sea, so that mismatch between plastic sinks and inputs can be eventually explained.

## Conclusions

Our results demonstrate the pervasiveness of plastic pollution in Mediterranean waters and, confirming model predictions, provide further evidence that in this basin, microplastic abundances are amongst the highest in the world. The Mediterranean Sea is the largest and deepest enclosed sea on earth, hot-spot of marine biodiversity and cradle of human civilization, whose unique outflow of water occurs at depth from the narrow Strait of Gibraltar. Being one of the busiest navigation crossroads and top touristic destinations in the world, surrounded by a heavily populated and industrialized coastline, it is not surprising that in this basin, the impacts of human activities are proportionally stronger than in any other sea^57^. Sinks, sources, fate and residence times of different polymers are the main knowledge gaps to be addressed, especially with regard to the smaller size classes. In addition, data from the eastern Mediterranean Sea are urgently needed, as numerical simulations predict preferential accumulation in the levantine basin. The polymeric characterization of plastic particles is of paramount importance for a proper assessment of plastic contamination in the marine environment and for the effective identification of specific solutions and alternatives. However, irrespective of different polymers sources and typologies, the problem of plastic pollution is a social and behavioural issue, whose causes require to be mostly sought upstream in the consumption chain.

## Methods

### Sampling and laboratory analys

74 neuston samples were collected during two consecutive cruises (CoCoPro13 and Venus2-ArgoIT) carried out in the Mediterranean Sea on board the Italian research vessel *Urania* between May 9th and June 24th 2013 (see Fig. S1 and Table S1 for details on sampling dates and locations). Samples were all collected using a Neuston net (100 × 50 cm rectangular frame opening) equipped with a 200 μm mesh size. At each station, the net was towed for ~5 minutes at a speed of 1.5–2 knots. All deployments were made from the ship’s starboard side beyond the bow wave, in order to avoid the wake turbulence. Once on board, the net was rinsed with seawater and the content of the cod end was emptied over a 50 μm metal sieve. Plankton samples were transferred to 50 ml falcon tubes, fixed with EtOH at a final concentration of 80% and stored for laboratory analysis. Sea surface temperature and salinity, were continuously recorded by the ship’s data logger and averaged over the duration of each tow. The length of the trawls (mean: 175.7 ± 85.5 m) was calculated using a mechanical flowmeter positioned at the center of the net mouth (Hydro-Bios).

In the laboratory, samples were examined under a dissecting stereo-microscope (Leica). Plastic particles were carefully hand-picked using laboratory tweezers and transferred to glass jars. All samples were double-checked by two different researchers to ensure detection of all particles and to reduce operator bias during sorting. Particles were then separated into seven size classes by sequential sieving through a series of stacked stainless steel meshes (0.2–0.3 mm, 0.3–0.5 mm, 0.5–0.7 mm, 0.7–1.0 mm, 1.0–2.8 mm, 2.8–5.0 mm and 5.0–20 mm). All particles in each size category were counted, dried in oven for 1 h at 50 °C, weighed on an electronic balance (accuracy: 0.1 mg) and classified according to five shape categories (industrial pellets, fragments, foam, thin films and filaments/fibers). The sampled area and the volume filtered during each trawl were calculated using the frame dimensions, the tow distance (derived from the flowmeter readings) and considering the net mouth as partially submerged in water for four-fifths (40 cm). Microplastic concentrations – expressed as mg/m^3^, g/km^2^, particles/m^3^ and particles/m^2^ for better comparison with previous studies – were then computed and plotted in Fig. 3 (See also Table S1 and Fig. S2). The examination of sample blanks during laboratory analysis, together with growing scientific evidence suggests that unless drastic precautions are taken, airborne contamination during sampling or processing is not negligible^58^. Hence, after being counted and classified, all filaments and fibers suspected of having a textile origin were excluded from density calculations.

### FT-IR analysis

In order to characterize the polymeric identity of the collected material, FT-IR (Fourier Transform Infrared Spectroscopy) analyses were performed on all particles >700 μm (n = 4,050). The subset was considered highly representative since it comprised 96.16% of the total weight of collected plastic. Analyses were performed using a Cary 660 FTIR spectrometer (Agilent) equipped with ATR (GladiATR Diamond Crystal Plate, Pike technologies) which allowed only the characterization of particles with grain size larger than 700 μm. Following background scans, 128 scans per particle were performed and CO_2_ interference (adsorption at approximately 2300–2400 cm^−1^) was removed for clarity. For each particle, scans were performed with a resolution of 4 cm^−1^. Agilent Resolution Pro v5.2 was used for the output spectra and identification of polymers was performed by comparison with a library of standard spectra. Only polymers matching reference spectra for more than 60% were accepted, in line with suggestions from previous studies^24^. Selected examples of output and reference spectra of the most common polymers found in our samples are shown in Fig. S4.

### Wind correction factor

Wind-driven mixing can extend the vertical distribution of buoyant particles below the sampling layer, therefore plastic concentrations were corrected using a widely used theoretical model^56^. Wind stress (*τ*) and significant wave height (*H*_*s*_) data were extracted from ECMWF ERA-Interim reanalysis product^59^, freely downloadable at http://apps.ecmwf.int/datasets/data/interim-full-daily/levtype=sfc/ (Date of access: 16/01/2016). Both fields were available with an original spatial resolution of 1/8° × 1/8° and a time frequency of 3 h and 6 h for *τ* and *H*_*s*_ respectively. The extracted data were linearly interpolated in time and space to each trawl date and location and conservatively incremented by 20% in order to compensate for well-known model underestimation in enclosed seas^60^. Friction velocities in water (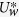) were derived from *τ* values assuming density of seawater *ρ*_*w*_ = 1.026 g/cm^3^. As suggested by the authors, wind correction was then applied only to net tows carried out with 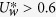 cm/s^56^ (*n* = 30; 40.5% of the tows), considering the immersion depth of the net (*d*) equal to 0.4 m and the buoyant rise velocity of plastics (*W*_*b*_) equal to 0.005 m/s^61^.

### Statistical analysis

Spearman’s correlation coefficient was used to test significant relationships between the abundance of floating particles and environmental variables, as well as between the abundance of different size classes. Spatial differences in the polymeric composition were highlighted through Principal Component Analysis (PCA) based on a variance-covariance matrix of the relative frequencies of the seven most common polymer classes: PP, PE, PS, PA, PVC, Nylon and Paint. Mann-Whitney U test was used to verify significant differences in plastic concentration and in the occurrence of the main polymer classes between sub-basins. Normality and homogeneity of variance was checked and the level of statistical significance was set at p < 0.05. Data analysis was performed using GraphPad Prism 6^©^ and PAST v3.11^62^.

## Additional Information

**How to cite this article**: Suaria, G. *et al*. The Mediterranean Plastic Soup: synthetic polymers in Mediterranean surface waters. *Sci. Rep.* **6**, 37551; doi: 10.1038/srep37551 (2016).

**Publisher's note:** Springer Nature remains neutral with regard to jurisdictional claims in published maps and institutional affiliations.

## Supplementary Material

Supplementary Material

## Figures and Tables

**Table 1:** Comparison of floating microplastic concentrations obtained in all previous studies performed in the Mediterranean Sea.

Study area	Sampling period	Net mesh	#	Mean abundance ± SD (Max)	Reference
Cretan Sea	July 1997	500 μm	25	119 ± 250 (1160) g/km^2^	63
NW Mediterranean	July–Aug 2010	333 μm	40	0.116 (0.892) items/m^2^	64
				2020 (2280) g/km^2^	
Ligurian/Sardinian Sea	Jun–Jul 2011	200 μm	23	0.31 ± 1.0 (4.83) items/m^2^	65
Bay of Calvi (Corsica)	Aug 2011–Aug 2012	200 μm	38	0.062 (0.688) items/m^2^	66
W Mediterranean	Sept 2011–Aug 2012	333 μm	41	0.135 (0.42) items/m^2^	55
				187 (216) g/km^2^	
W Sardinia	July 2012–July 2013	500 μm	30	0.15 (0.35) items/m^3^	67
Ligurian Sea	July–Aug 2013	333 μm	35	0.103 (0.36) items/m^2^	68
NW Sardinia	July 2012–July 2013	200 μm	27	0.17 ± 0.32 (1.69) items/m^3^	69
Ligurian Sea	Summer 2011/2012/2013	200 μm	70	0.31 ± 1.17 (9.67) items/m^3^	70
Mediterranean	May 2013	200 μm	39	0.243 items/m^2^	19
				423 (1934) g/km^2^	
Central W Mediterranean	May 2011–June 2013	333 μm	71	0.147 (1.16) items/m^2^	53
				579.3 (9298) g/km^2^	
W Med/Adriatic	May–June 2013	200 μm	74	0.40 ± 0.74 (4.52) items/m^2^	This study
				1.00 ± 1.84 (11.30) items/m^3^	
				671.91 ± 1544.16 (10432.36) g/km^2^	

For this study only concentrations of chemically characterized particles (>700 μm), un-corrected for the wind effect are shown. Some unit of measurements were converted to improve inter-comparability of the results.
