# Supplementary material for: The Mediterranean Plastic Soup: synthetic polymers in Mediterranean surface waters
Source: Sci Rep. 2016 Nov 23;6:37551. doi: 10.1038/srep37551 (PMC5120331; doi:10.1038/srep37551)
Supplement: Supplementary Material [file srep37551-s1.pdf]

Supplementary material

The Mediterranean Plastic Soup: synthetic polymers in Mediterranean surface waters

Giuseppe Suaria[1,2], Carlo G. Avio[3], Annabella Mineo[1], Gwendolyn L. Lattin[4], Marcello G. Magaldi[1,5], Genuario Belmonte[6], Charles J. Moore[4], Francesco Regoli[3,7], and Stefano Aliani[1,*]

[1] CNR-ISMAR, Pozzuolo di Lerici - La Spezia (Italy)
[2] Instituto Español de Oceanografía, Centro Oceanográfico de Baleares, Palma de Mallorca (Spain)
[3] Dipartimento di Scienze della Vita e dell'Ambiente (DiSVA), Universita Politecnica delle Marche, Ancona (Italy)
[4] Algalita Marine Research and Education, Long Beach, California (USA)
[5] Department of Earth and Planetary Sciences, Johns Hopkins University, Baltimore, MD (USA)
[6] Universita del Salento, DiSTeBA, Lecce (Italy)
[7] CoNISMa, Consorzio Interuniversitario per le Scienze del Mare, Roma (Italy)
[*] stefano.aliani@sp.ismar.cnr.it

Table S1 Date, GPS position, number and concentration of particles measured in all sampling stations. Synthetic fibers and filaments were excluded from density calculations. Only particles > 700 μm were chemically identified.

Station	Date	Latitude	Longitude	# Particles	Characterized Particles (> 700 μm)	Abundances							
						Particles/m^2			Particles/m^3			g/km^2	mg/m^3
						> 700 μm	< 700 μm	Total	> 700 μm	< 700 μm	Total	> 700 μm	
1	09/05/13	42,16650	16,16667	322	81	0,29	0,93	1,22	0,73	2,32	3,05	1484,85	3,712
2	09/05/13	42,10683	15,61517	158	75	0,20	0,46	0,67	0,51	1,16	1,67	1430,38	3,576
3	10/05/13	42,16683	16,63317	178	14	0,12	0,38	0,49	0,29	0,94	1,23	325,88	0,815
4	10/05/13	41,99917	16,99783	451	112	0,35	1,95	2,30	0,88	4,87	5,76	428,79	1,072
5	11/05/13	42,18017	18,83333	43	27	0,03	0,19	0,22	0,06	0,48	0,54	6,53	0,016
6	11/05/13	42,16650	18,53317	109	39	0,16	1,26	1,42	0,39	3,16	3,55	65,10	0,163
7	11/05/13	41,49983	18,36633	110	8	0,07	0,22	0,29	0,17	0,55	0,72	26,04	0,065
8	12/05/13	41,49950	18,83550	30	7	0,03	0,04	0,06	0,07	0,09	0,16	969,62	2,424
9	12/05/13	41,82483	19,21000	11	101	0,14	0,17	0,31	0,36	0,43	0,78	0,00	0,000
10	12/05/13	42,03717	19,00367	22	20	0,01	0,09	0,10	0,02	0,23	0,26	14,01	0,035
11	13/05/13	41,03833	18,53333	101	39	0,06	0,47	0,52	0,14	1,16	1,30	47,03	0,118
12	13/05/13	40,88450	18,95467	324	41	0,31	1,30	1,60	0,77	3,24	4,01	287,27	0,718
13	13/05/13	40,44950	19,13283	119	32	0,15	0,33	0,48	0,37	0,82	1,19	120,34	0,301
14	13/05/13	40,50383	19,22667	21	55	0,06	0,07	0,14	0,16	0,18	0,34	38,61	0,097
15	14/05/13	40,30717	19,33867	145	65	0,13	0,32	0,44	0,32	0,79	1,11	33,67	0,084
16	14/05/13	40,17200	19,39333	95	13	0,04	0,61	0,65	0,10	1,51	1,62	27,21	0,068
17	14/05/13	40,12650	19,30850	137	16	0,11	0,58	0,69	0,28	1,44	1,71	45,05	0,113
18	14/05/13	40,08333	19,13333	33	11	0,16	0,24	0,40	0,40	0,61	1,01	48,84	0,122
19	15/05/13	39,66667	19,36667	1187	18	2,51	2,15	4,65	6,27	5,36	11,64	5917,65	14,794
20	15/05/13	39,66650	19,13217	46	28	0,12	0,05	0,17	0,30	0,12	0,42	339,91	0,850
21	16/05/13	39,66650	18,36633	77	19	0,06	0,28	0,34	0,14	0,70	0,84	46,13	0,115
22	16/05/13	40,08333	18,83400	68	9	0,07	0,31	0,38	0,18	0,77	0,95	5,57	0,014
23	16/05/13	40,23117	18,50617	50	47	0,07	0,33	0,41	0,18	0,83	1,02	178,86	0,447
24	17/05/13	40,45050	18,53317	82	7	0,05	0,26	0,31	0,13	0,65	0,79	49,81	0,125
25	17/05/13	41,03800	17,86683	134	4	0,13	0,26	0,40	0,33	0,66	1,00	65,42	0,164
26	17/05/13	41,03833	17,58400	582	55	0,75	2,45	3,21	1,89	6,13	8,02	280,99	0,702
27	18/05/13	41,49950	16,91683	38	188	0,08	0,15	0,24	0,20	0,39	0,59	6,20	0,015
28	18/05/13	41,50267	17,54883	10	81	0,02	0,02	0,04	0,05	0,05	0,09	0,00	0,000
29	17/05/13	40,76117	17,78333	84	36	0,05	0,26	0,31	0,13	0,65	0,78	11,20	0,028
30	05/06/13	39,33380	17,82005	396	21	0,41	1,96	2,37	1,03	4,90	5,94	1732,61	4,332
31	08/06/13	37,31400	11,56300	390	285	0,53	1,68	2,22	1,34	4,21	5,55	193,40	0,484
32	08/06/13	37,41600	11,74400	66	625	0,08	0,34	0,42	0,19	0,85	1,04	69,58	0,174
33	08/06/13	37,45900	11,83000	278	70	0,62	0,65	1,28	1,56	1,63	3,19	2286,50	5,716
34	08/06/13	37,58700	12,00600	91	119	0,12	0,38	0,49	0,30	0,94	1,24	54,38	0,136
35	08/06/13	37,64800	12,14500	99	50	0,18	0,28	0,46	0,44	0,70	1,14	59,94	0,150
36	08/06/13	37,72900	12,33500	25	26	0,01	0,10	0,11	0,03	0,25	0,28	4,52	0,011
37	08/06/13	38,08600	11,95700	27	480	0,05	0,13	0,18	0,12	0,34	0,45	40,40	0,101
38	08/06/13	38,12000	11,84600	264	12	0,66	1,06	1,72	1,64	2,65	4,29	571,80	1,429
39	08/06/13	38,18100	11,66700	66	46	0,15	0,26	0,41	0,37	0,65	1,03	105,92	0,265
40	08/06/13	38,23400	11,53100	203	6	0,23	0,92	1,15	0,57	2,31	2,88	124,72	0,312
41	09/06/13	38,53400	10,86900	124	74	0,22	0,57	0,79	0,56	1,41	1,97	4234,92	10,587
42	09/06/13	38,63200	10,68400	123	50	0,16	0,56	0,71	0,39	1,39	1,78	34,78	0,087
43	09/06/13	38,71110	10,47000	110	50	0,37	0,28	0,65	0,92	0,71	1,63	248,67	0,622
44	09/06/13	38,80500	10,25800	159	37	0,30	0,53	0,83	0,74	1,33	2,07	176,81	0,442
45	09/06/13	38,85700	10,18300	53	63	0,09	0,25	0,34	0,23	0,63	0,86	32,36	0,081
46	09/06/13	38,91400	10,01500	76	132	0,15	0,78	0,92	0,36	1,95	2,31	48,66	0,122
47	09/06/13	39,01400	9,78700	88	31	0,20	1,15	1,35	0,50	2,88	3,38	61,44	0,154
48	11/06/13	38,19171	8,80000	51	30	0,08	0,25	0,33	0,19	0,63	0,82	290,70	0,727
49	11/06/13	38,20100	7,81300	92	46	0,10	0,37	0,47	0,26	0,92	1,18	440,35	1,101
50	12/06/13	38,15500	7,26300	51	6	0,11	0,44	0,55	0,27	1,11	1,38	32,47	0,081
51	12/06/13	38,11600	6,63700	34	22	0,06	0,30	0,37	0,16	0,76	0,92	32,47	0,081
52	12/06/13	38,06900	5,96900	142	25	0,72	1,46	2,18	1,80	3,65	5,45	1950,84	4,877
53	12/06/13	38,03100	5,32400	24	12	0,12	0,28	0,40	0,29	0,70	1,00	49,75	0,124
54	13/06/13	37,97800	4,64700	64	9	0,15	2,25	2,40	0,37	5,62	5,99	0,00	0,000
55	13/06/13	39,80334	4,40396	160	13	0,39	0,66	1,05	0,97	1,66	2,62	360,89	0,902
56	13/06/13	39,80334	4,60778	524	29	2,65	4,26	6,90	6,62	10,64	17,26	2279,31	5,698
57	15/06/13	39,77417	11,88833	229	42	0,45	0,75	1,20	1,12	1,87	2,99	407,52	1,019
58	16/06/13	43,02500	9,70000	74	34	0,21	0,42	0,64	0,54	1,05	1,59	463,92	1,160
59	16/06/13	43,02517	9,64167	53	7	0,09	0,25	0,34	0,22	0,62	0,84	304,76	0,762
60	17/06/13	43,02600	9,59967	850	15	1,62	2,22	3,84	4,04	5,56	9,60	1531,17	3,828
61	17/06/13	43,02617	9,52533	1324	7	4,52	4,71	9,23	11,30	11,79	23,08	10432,36	26,081
62	17/06/13	43,03002	9,76987	281	16	0,39	1,21	1,60	0,97	3,03	4,00	335,61	0,839
63	19/06/13	39,80334	7,99863	371	6	1,96	3,54	5,50	4,89	8,85	13,74	2957,04	7,393
64	19/06/13	39,80067	7,81933	196	74	0,42	2,07	2,48	1,05	5,16	6,21	139,42	0,349
65	20/06/13	39,80017	7,39950	112	30	0,32	0,80	1,12	0,80	2,00	2,79	538,92	1,347
66	20/06/13	39,80067	6,99983	944	8	2,55	2,49	5,04	6,37	6,24	12,61	3039,53	7,599
67	20/06/13	39,79933	6,61000	34	14	0,18	0,69	0,87	0,45	1,73	2,18	128,21	0,321
68	20/06/13	39,80334	5,98607	230	7	0,47	1,80	2,27	1,19	4,50	5,69	276,95	0,692
69	20/06/13	39,80050	5,40950	53	10	0,12	0,66	0,77	0,29	1,64	1,93	291,12	0,728
70	21/06/13	39,80334	4,81302	111	28	0,38	0,33	0,71	0,96	0,81	1,77	376,76	0,942
71	22/06/13	38,32400	9,30300	240	99	0,15	0,87	1,02	0,37	2,17	2,54	38,12	0,095
72	22/06/13	38,37970	9,52133	118	7	0,24	0,46	0,70	0,61	1,14	1,75	130,25	0,326
73	22/06/13	38,54450	9,82533	122	22	0,21	0,68	0,89	0,53	1,70	2,23	175,82	0,440
74	24/06/13	38,91650	13,29933	217	7	0,18	0,75	0,93	0,45	1,88	2,33	334,62	0,837
			**Mean**	**190,62**	**54,73**	**0,40**	**0,85**	**1,25**	**1,00**	**2,13**	**3,13**	**671,91**	**1,68**

Fig. S1 Map of the study area showing the location of all sampling stations numbered in sequential order. Data were plotted using GPS Visualizer (http://www.gpsvisualizer.com) and post-edited in Adobe Illustrator CS5. Background map freely retrieved from DEMIS OpenGIS Web Map Server under open copyright licence (http://www.demis.nl/home/pages/wms/docs/OpenGISWMS.htm).

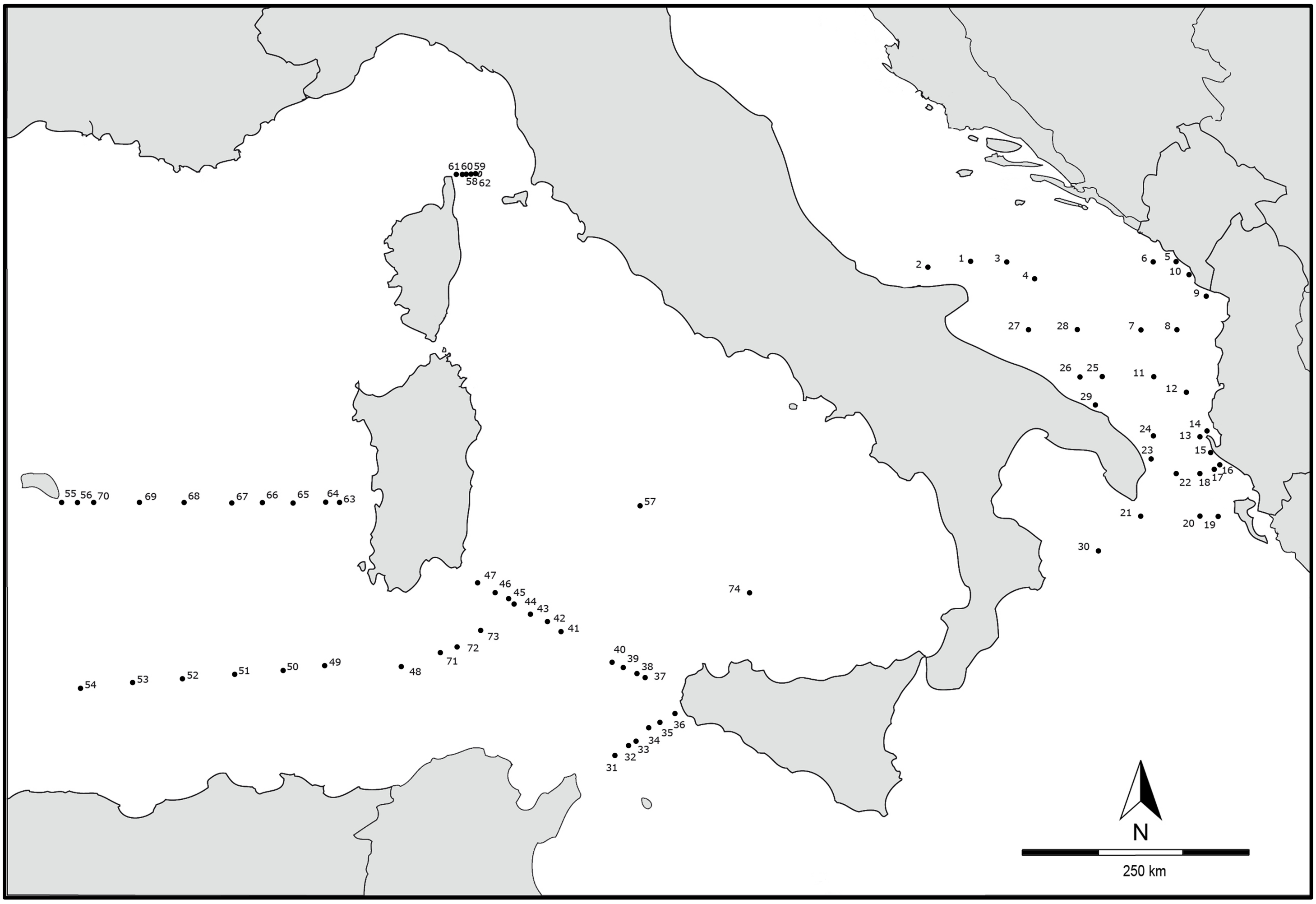

Fig. S2 Cumulative concentration of particles bigger and smaller than 700 µm measured in all sampling stations.

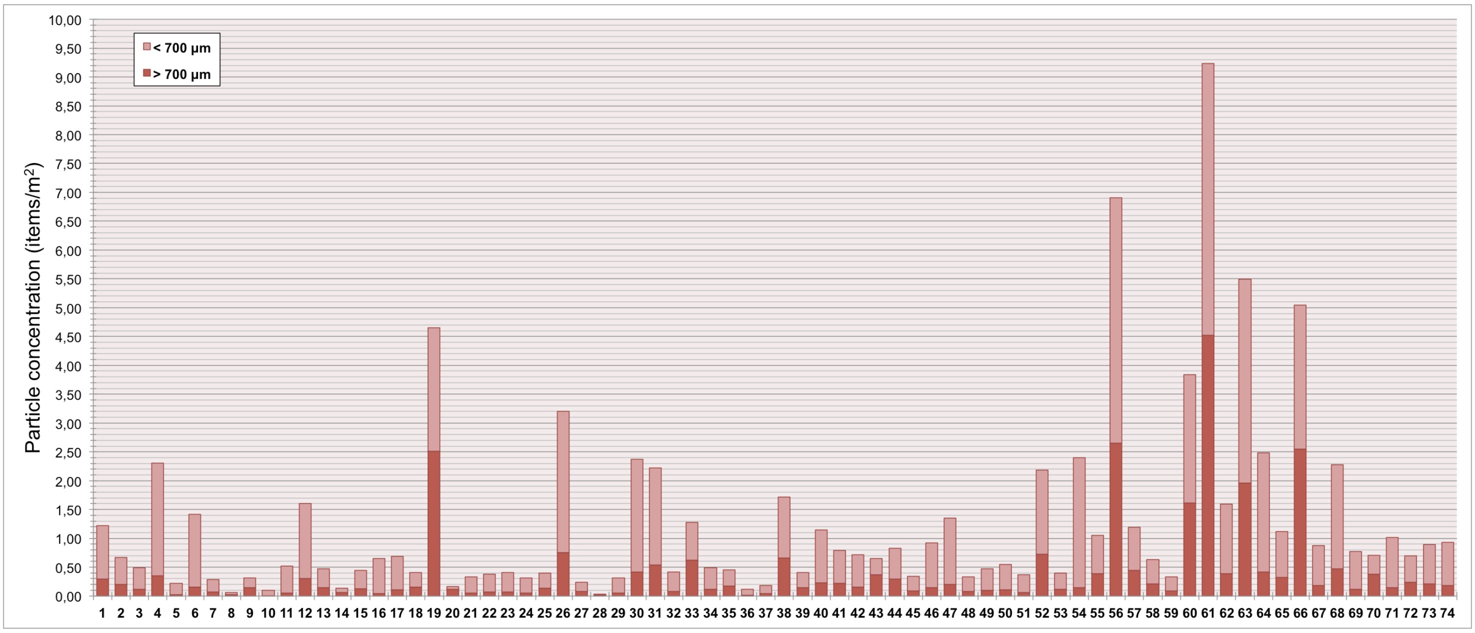

Fig. S3 FT-IR analysis results. Polymeric composition of all particles > 700 µm collected in each sampling station.

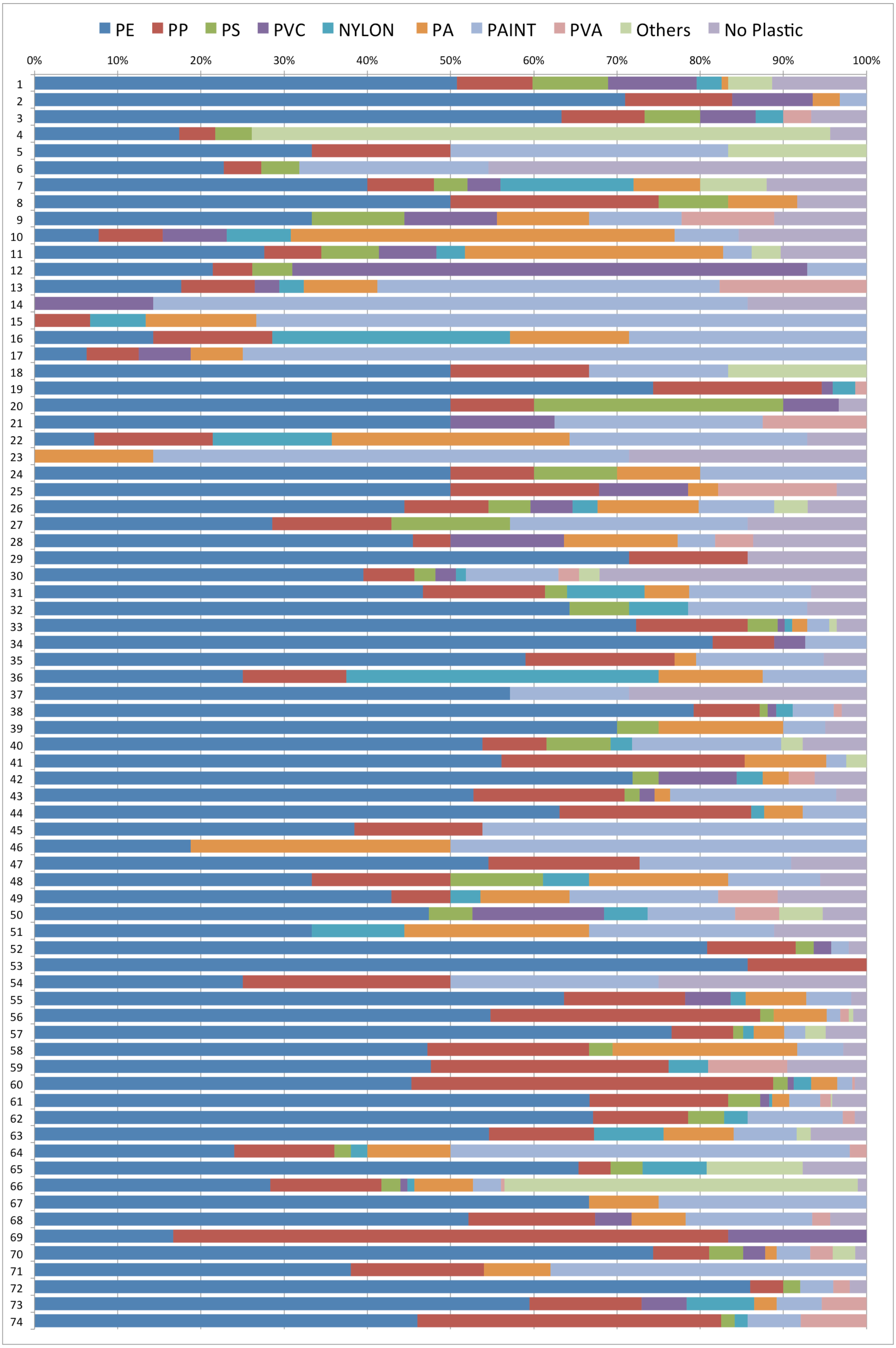

Fig. S4. Examples of Fourier transform infrared (FT-IR) spectra from plastic particles collected in Mediterranean surface waters. Spectra of plastic particles are shown in green and red, reference spectra (from reference library) are shown in blue.

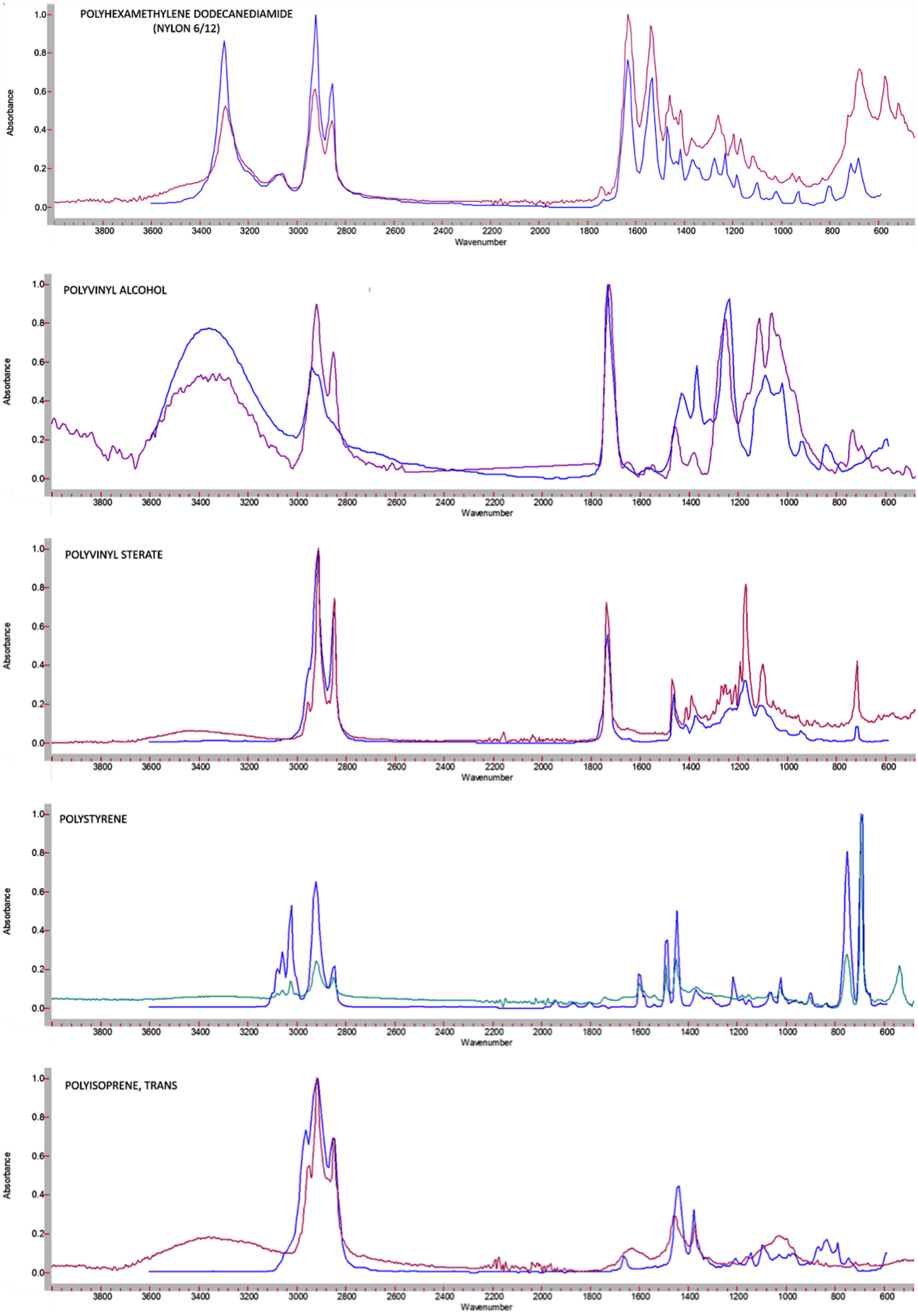

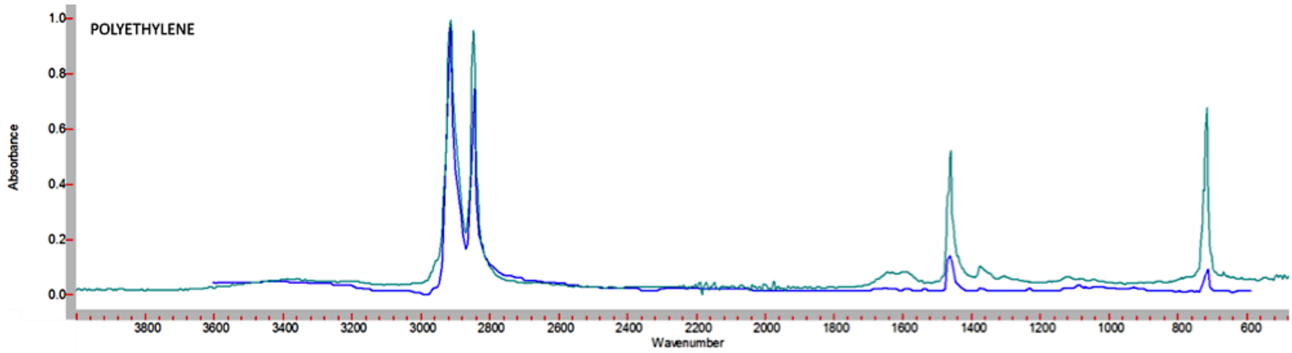
POLYETHYLENE
Absorbance
1.0
0.8
0.6
0.4
0.2
0.0
3800 3600 3400 3200 3000 2800 2600 2400 2200 2000 1800 1600 1400 1200 1000 800 600
Wavenumber

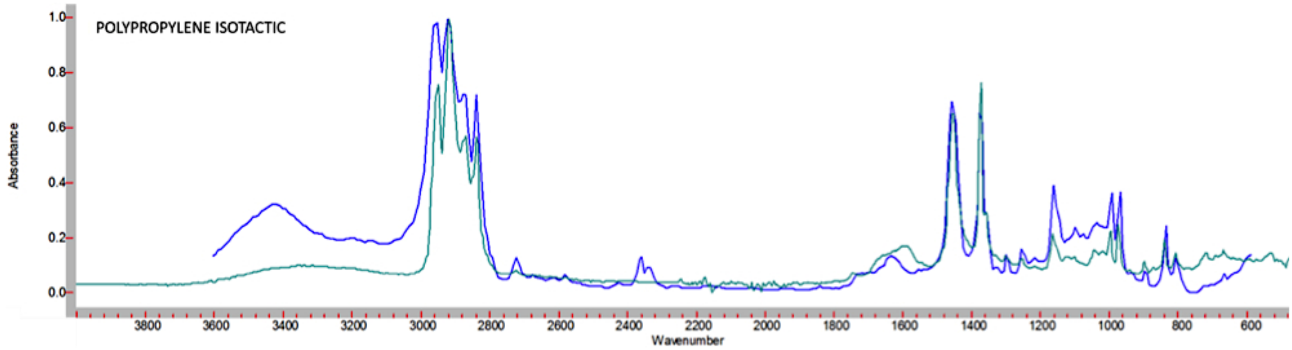
POLYPROPYLENE ISOTACTIC
Absorbance
1.0
0.8
0.6
0.4
0.2
0.0
3800 3600 3400 3200 3000 2800 2600 2400 2200 2000 1800 1600 1400 1200 1000 800 600
Wavenumber

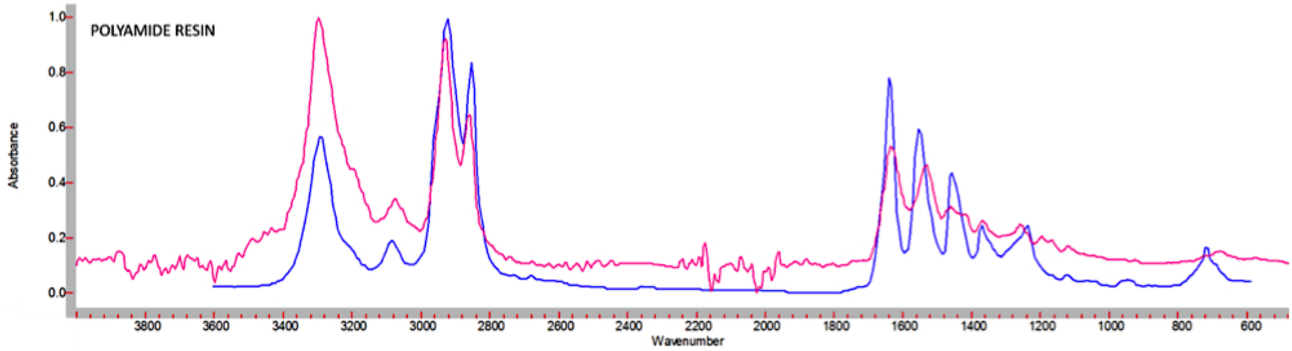
POLYAMIDE RESIN
Absorbance
1.0
0.8
0.6
0.4
0.2
0.0
3800 3600 3400 3200 3000 2800 2600 2400 2200 2000 1800 1600 1400 1200 1000 800 600
Wavenumber

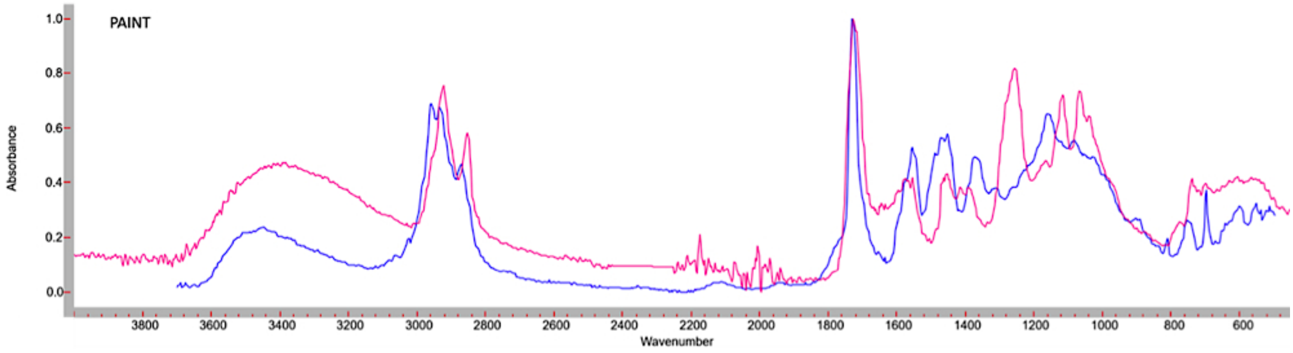
PAINT
Absorbance
1.0
0.8
0.6
0.4
0.2
0.0
3800 3600 3400 3200 3000 2800 2600 2400 2200 2000 1800 1600 1400 1200 1000 800 600
Wavenumber

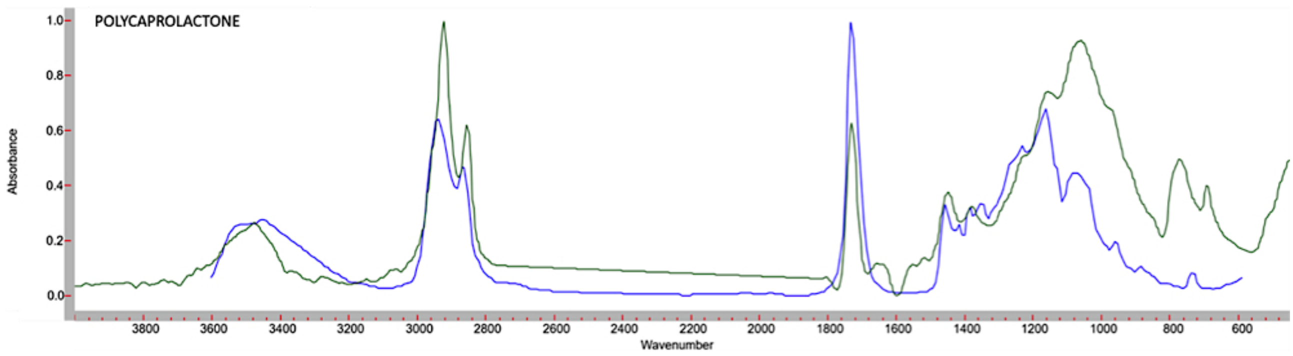
POLYCAPROLACTONE
Absorbance
1.0
0.8
0.6
0.4
0.2
0.0
3800 3600 3400 3200 3000 2800 2600 2400 2200 2000 1800 1600 1400 1200 1000 800 600
Wavenumber
